# Contrast-Enhanced Harmonic Endoscopic Ultrasonography for Prediction of Aggressiveness and Treatment Response in Patients with Pancreatic Lesions

**DOI:** 10.3390/cancers17152545

**Published:** 2025-08-01

**Authors:** Marco Spadaccini, Gianluca Franchellucci, Marta Andreozzi, Maria Terrin, Matteo Tacelli, Piera Zaccari, Maria Chiara Petrone, Gaetano Lauri, Matteo Colombo, Valeria Poletti, Giacomo Marcozzi, Antonella Durante, Roberto Leone, Maria Margherita Massaro, Antonio Facciorusso, Alessandro Fugazza, Alessandro Repici, Paolo Giorgio Arcidiacono, Silvia Carrara

**Affiliations:** 1 Department of Biomedical Sciences, Humanitas University, 20072 Pieve Emanuele, Italy; marta.andreozzi@humanitas.it (M.A.); maria.terrin@humanitas.it (M.T.); matteo.colombo@humanitas.it (M.C.); valeria.poletti@humanitas.it (V.P.); giacomo.marcozzi@humanitas.it (G.M.); antonella.durante@humanitas.it (A.D.); roberto.leone@humanitas.it (R.L.); mariamargherita.massaro@humanitas.it (M.M.M.); alessandro.repici@hunimed.eu (A.R.); silvia.carrara@humanitas.it (S.C.); 2 Endoscopy Unit, Humanitas Clinical and Research Center—IRCCS, 20089 Rozzano, Italy; gianluca.franchellucci@humanitas.it (G.F.); alessandro.fugazza@humanitas.it (A.F.); 3 Pancreato-Biliary Endoscopy and Endosonography Division, Pancreas Translational and Clinical Research Center, IRCCS San Raffaele Scientific Institute, 20132 Milan, Italy; tacelli.matteo@hsr.it (M.T.); zaccari.piera@hsr.it (P.Z.); petrone.mariachiara@hsr.it (M.C.P.); lauri.gaetano@hsr.it (G.L.); arcidiacono.paologiorgio@hsr.it (P.G.A.); 4 Faculty of Medicine and Surgery, University of Salento, 73100 Lecce, Italy; antonio.facciorusso@virgilio.it

**Keywords:** NET, IPMN, CH-EUS, aggressiveness, PDAC, vascularity, microcirculation, prognosis, chemotherapy, surgery

## Abstract

Endoscopic ultrasonography represents a crucial aspect of the diagnosis of pancreatic lesions. The echo-endoscopic features of pancreatic lesions, particularly their contrast behavior with the advent of Contrast-Enhanced EUS (CE-EUS) and Contrast Enhanced Harmonic-EUS (CH-EUS), can predict a lesion’s aggressiveness, depending on its nature. According to this, CH-EUS could be applied to structure an even more dedicated approach to patient care, for example, to ascertain eligibility for surgical intervention of a pancreatic ductal adenocarcinoma (PDAC) or the response to neoadjuvant chemotherapy in cases deemed borderline resectable. In addition to PDAC, other significant issues pertain to the management of small neuroendocrine tumors (NETs) and intraductal papillary mucinous neoplasms (IPMNs). In this context, CH-EUS can be crucial. The aim of this review is to underline the most recent evidence for EUS and CH-EUS applications in pancreatic lesion aggressiveness assessment and to focus on possible future research directions to further extend the application of CH-EUS in this field.

## Simple Summary

Endoscopic ultrasonography (EUS) is essential for diagnosing pancreatic lesions, with recent advancements like Contrast-Enhanced EUS (CE-EUS) and Contrast-Enhanced Harmonic EUS (CH-EUS) enhancing the ability to assess lesion aggressiveness. CH-EUS, in particular, helps guide clinical decisions such as determining surgical eligibility for pancreatic ductal adenocarcinoma (PDAC) or evaluating response to neoadjuvant chemotherapy in borderline resectable cases. It also plays a key role in managing small neuroendocrine tumors (NETs) and intraductal papillary mucinous neoplasms (IPMNs). This review summarizes current evidence on EUS and CH-EUS in evaluating pancreatic lesions and emphasizes the potential for future research to expand CH-EUS applications in personalized patient management.

## 1. Introduction

Endoscopic ultrasound (EUS) is, to date, the preferred method for the detection and characterization of pancreatic lesions due to its high sensitivity and the possibility of acquiring tissue samples [1].

**Figure 1:**
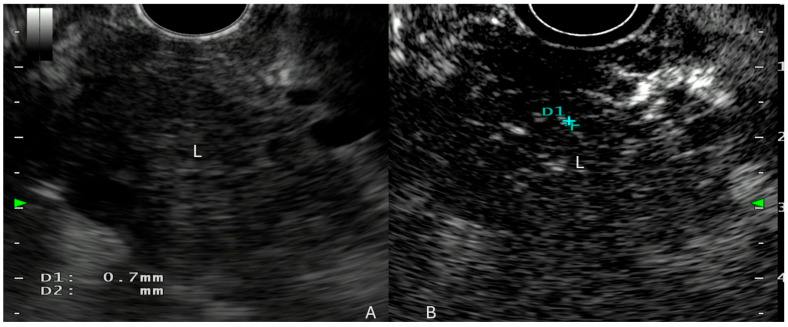
Pancreatic adenocarcinoma. (**A**): lesion (L) on EUS vision; (**B**): hypoenhancement of the lesion after Sonouvue infusion. D1: intralesional vessel.

In this context, contrast-enhanced harmonic endoscopic ultrasonography (CH-EUS) has demonstrated particular value in pancreatic ductal adenocarcinoma (PDAC) (REF), one of the most aggressive and fatal cancers globally [2]. Compared to traditional imaging modalities such as CT and MRI, CH-EUS has shown superior diagnostic and staging accuracy [3,4,5]. A meta-analysis revealed that CH-EUS significantly improves the detection of pancreatic malignancies, with diagnostic odds ratios notably higher than those achieved with standard EUS [6] (Figure 1). Beyond initial diagnosis, CH-EUS enhances the precision of preoperative staging, especially in evaluating vascular invasion—a critical determinant of surgical resectability. For example, studies comparing CH-EUS with contrast-enhanced CT for detecting portal vein invasion have consistently demonstrated the superior diagnostic accuracy of CH-EUS, highlighting its importance in managing borderline resectable cases [7]. While CH-EUS provides unparalleled imaging and diagnostic capabilities, EUS-guided tissue acquisition (FNA/FNB) remains essential for histopathological confirmation. The use of contrast agents during EUS-FNA has been shown to enhance diagnostic yield by targeting areas with subtle vascularization, ensuring precise sampling [8]. This synergistic integration of advanced imaging and tissue acquisition reinforces the central role of CH-EUS in the comprehensive management of pancreatic lesions. Moreover, CH-EUS plays a pivotal role in the evaluation of small pancreatic neuroendocrine tumors (pNETs) and intraductal papillary mucinous neoplasms (IPMNs). For low-grade pNETs, CH-EUS provides valuable information that can guide the decision between immediate surgical intervention and a conservative “watch-and-wait” approach. Similarly, in IPMNs, CH-EUS aids in identifying features suggestive of malignant transformation, enabling clinicians to stratify risk and tailor management plans accordingly [9,10].

While EUS has historically played a central role in diagnostic workflows for pancreatic conditions, recent advancements have underscored its potential as a prognostic tool. In particular, contrast-enhanced harmonic endoscopic ultrasonography (CH-EUS) has emerged as a transformative modality, leveraging contrast patterns to reveal vascular and structural features that provide crucial insights into tumor aggressiveness and treatment response [4,11]. This capability enables clinicians to make informed decisions, enhancing patient outcomes through personalized management strategies.

This review aims to explore the potential of CH-EUS in assessing the risk features of IPMNs, predicting solid lesion aggressiveness, and assessing pathological responses to chemotherapy in patients with pancreatic tumors. By examining its application in IPMN, PDAC, and pNETs, we aim to highlight how CH-EUS is reshaping the diagnostic and therapeutic landscape, offering a bridge between imaging precision and personalized treatment approaches.

## 2. EUS and CH-EUS

By the end of the previous century, endoscopic ultrasound (EUS) had become the preferred modality for detecting and characterizing pancreatic lesions. This can be attributed to its superior sensitivity and specificity compared to computed tomography (CT) and magnetic resonance imaging (MRI), especially for diagnosing small pancreatic lesions (≤2 cm) [3,4,5].

A significant advancement in EUS came with the integration of Doppler techniques, followed by the administration of contrast agents, which have been shown to enhance the sensitivity and specificity for detecting pancreatic lesions [12]. However, it should be noted that the Doppler signal, whether used with or without contrast agents, can result in a high rate of artifacts during vascularity assessment. On the other hand, contrast-enhanced EUS (CE-EUS) has been found to have limitations in accurately depicting microvascularity within tissue [13].

An advancement in the technique is contrast-enhanced harmonic EUS (CH-EUS), which involves a dedicated endosonographic imaging modality beyond standard Doppler. CH-EUS is particularly useful for minimizing artifacts and providing detailed visualization of microvascularity, made possible by the advent of second-generation EUS contrast agents [12,14,15]. These agents consist of microbubbles encased in a lipid shell, which are selectively detected by harmonic imaging. This allows for the identification of vessels with very slow flow and provides more accurate imaging of tissue microcirculation. They mimic the contrast phases typically observed in CT and MRI: the arterial phase occurs 15–30 s post-injection, the portal vein phase at 30–45 s, and the late phase at 120 s. Three of them are currently available: Sonouve (Bracco Imaging, Milan, Italy), Sonazoid (Daiichi Sankyo, Tokyo, Japan; GE Healthcare, Oslo, Norway), and Definity (Lantheus Medical Imaging, N. Billerica, MA, USA). Notably, Sonazoid exhibits a special feature called the Kupffer phase, which results from the engulfing of lipid shells by liver Kupffer cells, allowing prolonged imaging time after the late phase [16,17,18].

CH-EUS has shown high sensitivity and specificity for diagnosing pancreatic lesions. A meta-analysis of over 800 patients demonstrated a sensitivity greater than 93% and specificity near 80% [19]. Different types of pancreatic lesions exhibit distinct patterns on CH-EUS. For example, pancreatic cancer (PC) typically shows diffuse hypoenhancement in the arterial phase [16,20], whereas pancreatic neuroendocrine tumors (pNETs) tend to have a hypervascular pattern [21].

One inherent limitation of CH-EUS—and endosonography in general—is its operator dependency. To help minimize this issue, a further evolution of CH-EUS is dynamic contrast-enhanced ultrasound (DCE-US). DCE-US is a functional imaging technique that allows quantitative estimation of the perfusion of a mass using raw linear data and calculation of objective parameters describing its vasculature [22]. Time intensity curves (TICs) are DCE parameters that measure real-time change in echo enhancement intensity, already used in the US in other districts [23]. The TIC is generated by selecting a region of interest (ROI) within the image and plotting the ultrasound signal intensity (brightness) against time. The resulting curve provides key parameters such as time to peak (TTP)—the time it takes for the contrast intensity to reach its maximum—peak intensity (PI) —the highest level of enhancement—and wash-in and wash-out slopes, indicating how quickly the contrast enters and leaves the tissue. These parameters help differentiate between benign and malignant lesions based on their vascular characteristics. For example, malignant tumors often show impaired wash-in and wash-out due to abnormal angiogenesis, while benign lesions may exhibit more uniform enhancement. TIC analysis aims to enhance the objectivity of CH-EUS by reducing operator dependency and providing reproducible, quantifiable data. It complements the qualitative assessment of vascularity and supports clinical decision-making. A 2017 study showed that PDAC, compared with pNENs, had significantly lower TICs values of peak intensity and the intensity at 60 s after contrast injection [24].

In recent years, Detective Flow Imaging (DFI-EUS), a new technology that does not require contrast agents, has been introduced. DFI-EUS allows dynamic visualization of blood flow at low speeds and high frame rates, with a lower detection threshold than conventional Doppler methods. This technique provides high-resolution and sensitive perfusion information, offering improved results compared to traditional Doppler methods [25,26].

DFI-EUS also has the advantage of being safe for patients for whom contrast agents are contraindicated and does not require adherence to strict temporal evaluation since contrast media can only be visualized within a specific time window [27]. The use of DFI in the evaluation of pancreatic-biliary lesions was evaluated in two studies in 2024: one by Yamashita et al., which, however, included only one NEN in the case cohort [28] and the second by Mulqui et al. in which the DFI-EUS showed good correlation with the reference methodology of vascular pattern assessment (CH-EUS) in solid pancreatic lesions; moreover, the cohort included 18 cases of pNEN, with DFI-EUS having sensitivity, PPV and NPV specificity for diagnosis of 94.1%, 100.0%, 100.0%, and 50%, respectively [28]. The limitation of using DFI compared to CH-EUS remains the absence of information based on the dynamic perfusion of the tissue.

## 3. CH-EUS in Pancreatic Cancer: Tumor Aggressiveness and Response to Chemotherapy

According to histological reports, it is known how cancer vascularity and stromal composition represent a clear and well-known prognostic aspect [29]. Tumors exhibiting well-developed intratumoral vascularity are generally more aggressive, while a higher percentage of fibrosis is associated with a reduced likelihood of metastasis [30].

Various imaging modalities such as CT, MRI, and CE-EUS have been used to assess tumor vascularity and stroma. Since 2010, CH-EUS has been intensively used in clinical practice for vascular assessment of pancreatic lesions. In fact, CH-EUS has a higher sensitivity and specificity for defining intratumoral vascularity, with the ability to detect even very small vessels down to 1 mm in diameter [31,32].

A 2005 study evaluating CE-EUS found that patients with avascular, unresectable pancreatic cancer had a significantly longer survival (267 days) compared to those with vascularized tumors (115 days) (*p* = 0.0034) [33]. In contrast, Sofouni et al.’s study involving 34 patients with advanced PC demonstrated that tumors with higher vascularity on CE-EUS correlated with a better response to chemotherapy and longer survival (402 days vs. 246 days) [11].

In a pilot study on patients with inoperable PC, 39 patients underwent CE-EUS before chemotherapy. The patients were divided into 2 groups according to the intratumoral vessel flow observed with CE-EUS: vessel sign-positive and vessel sign-negative groups. Both progression-free survival (PFS) and overall survival (OS) were significantly longer in the positive versus negative vessel sign groups (*p* = 0.037 and *p* = 0.027, respectively). Multivariate analysis demonstrated that the positive vessel sign was an independent factor associated with longer OS (HR, 0.22; 95% confidence interval, 0.08–0.53) [34].

Also, CH-EUS was investigated to predict chemotherapy response of PDAC at baseline. Emori and colleagues analysed chemotherapy response based on the enhancement patterns of PDAC during both the arterial phase (10–30 s after contrast injection) and the venous phase (30–120 s after contrast injection). The pancreatic lesions were categorized into four enhancement patterns: Group A (no vascularity in both phases), Group B (rich vascularity only in the early phase), Group C (rich vascularity only in the late phase), and Group D (rich vascularity in both phases). The study focused on patients with unresectable locally advanced or metastatic PDAC and assessed their chemotherapy responses based on the enhancement pattern. Patients in Group D, who had rich vascularity in both phases, demonstrated a higher rate of tumor reduction, biological tumor response, and better PFS and OS [35].

Finally, CH-EUS has also been used not only to predict but also to assess and quantify chemotherapy response in PC. In a 2019 study, 21 patients with unresectable PC were divided into two groups after a cycle of chemotherapy based on their CA 19.9 response. Patients who showed a drop in CA 19.9 levels of more than 50% after chemotherapy were classified as “super responders.” The study found that super responders had a higher frequency of avascular areas on CH-EUS, with 7 out of 9 (77.8%) showing this feature, compared to only 4 out of 14 (28.6%) in patients who did not experience a similar drop in CA 19.9 levels [36].

## 4. CH-EUS in Intraductal Papillary Mucinous Neoplasm (IPMN)

Intraductal papillary mucinous neoplasm (IPMN) is a prevalent pancreatic lesion that is frequently encountered during clinical practice [37,38]. The diagnosis and characterization of IPMN are of crucial importance, as these lesions have the potential to develop into a malignant invasive disease [39]. The morphological features of IPMN are fundamental to define the probability of malignancy [37,38,39]. As stated in various guidelines, the features considered as “worrisome” or “high-risk” stigmata include cyst dimensions, the presence of mural nodules and their dimensions, pancreatic duct dilation, thickness of the cystic walls, the rate of cyst growth, the presence of lymphadenopathies and variation of the pancreatic duct with related pancreatic atrophy [37,38,40,41,42]. In this context, EUS has shown higher effectiveness than CT scan and MRI in detecting morphological features suggestive of IPMN progression, as evidenced by a comprehensive body of research [43,44,45,46,47,48,49]. As a consequence, EUS has gained a prominent role in the assessment of worrisome and high-risk stigmata, as also stated in the recently published Kyoto guidelines [37,50,51,52].

**Figure 2:**
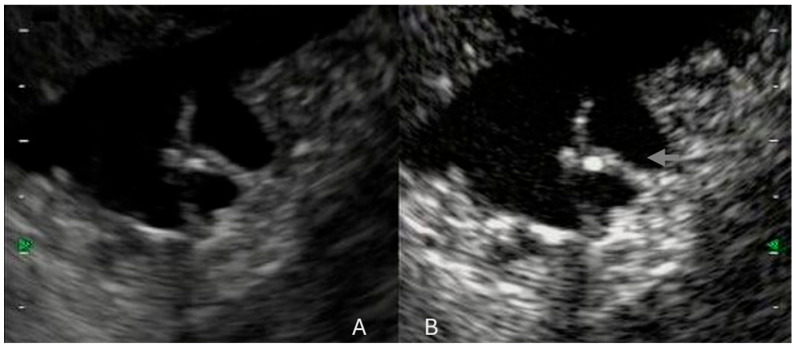
IPMN visualization on EUS (**A**) and on CH-EUS (**B**). (**B**) IPMN evaluation with CH-EUS using Sonouvue. Gray arrow: Hyperenhancement of the intralesional septum.

CH-EUS has also given improvements in this field [53]. The first use of contrast in EUS studies for IPMNs was to help distinguish between mucous lumps and mural nodules. Both can appear similar on regular (B-mode) EUS, which makes it challenging to tell them apart. Mural nodules often show different patterns of enhancement compared to the surrounding cystic fluid. In contrast, mucous lumps generally have less blood flow and may not enhance as much [54,55] (Figure 2).

In 2016, Kamata et al. demonstrated that CH-EUS was superior to B-mode EUS for detecting mural nodules [35,56]. This was an initial experience with a limited sample size (70 patients), including both mucinous cysts and IPMN. It was then confirmed by a subsequent analysis on 115 cases of IPMNs performed by Yamashita et al., who confirmed the better performance of CH-EUS compared to B-mode EUS and CE-CT scan in mural nodule detection [57]. In the same study, the authors interactively compared the qualitative pattern of CH-EUS with histological reports. Early contrast wash-out of the lesions was associated with IPMN with high-grade dysplasia or intraductal papillary mucinous carcinoma. Specifically, an early wash-out pattern on CH-EUS was detected in 22 out of 38 (58%) cases of invasive IPMC, while this pattern was not observed in LGD/IGD (0/31) or HGD (0/46) [57].

In 2009, a retrospective analysis on 87 patients describing the predictors of malignancy of IPMNs using CH-EUS was published. The authors classified mural nodules, defined as blood flow-supplied protrusions, into four patterns: type I (low papillary nodule), a low protruding component in the cyst wall or in the central pancreatic duct (MPD); type II (polypoid nodule), a smooth-surfaced component protruding into the cyst or MPD; type III (papillary nodule), a protruding component with a thickened cyst wall or MPD epithelium or with an irregular, villous structure; and type IV (invasive nodule), a lesion in which papillary nodules were connected to a hypoechoic area ill-defined from the pancreatic parenchyma. In multivariate analysis, types III and IV were related to malignancy (odds ratio 10.8; 95% CI: 2.75–56.1), with a sensitivity, specificity, and accuracy of 60%, 92.9%, and 75.9%, respectively [58].

**Figure 3:**
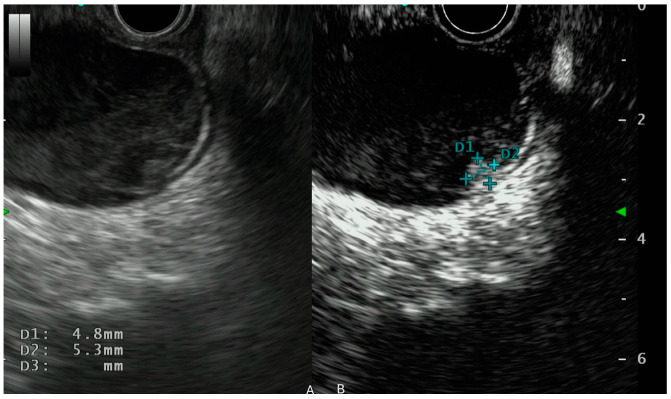
IPMN with a mural nodule, visualization on EUS (**A**) and on CH-EUS (**B**). (**B**) IPMN evaluation with CH-EUS using Sonouvue. Hyperenhancement of the mural nodule. D1 and D2 indicate the diameter of the nodule.

In a further significant experience in this field, Yamamoto et al. also correlated the variation of the time–intensity curve in CH-EUS with the IPMNs’ malignancy. The authors divided 40 surgically resected cases into two groups: those with LGD or IGD were classified as benign, while those with HGD or invasive carcinoma were classified as malignant. The authors then compared histology with the parameters of the time–intensity curve variation. The study revealed that among the parameters examined, including echo intensity change, echo intensity reduction rate, and nodule/pancreatic parenchyma contrast ratio, IPMNs associated with HGD/invasive carcinoma exhibited significantly higher values compared to those associated with LGD/IGD. Additionally, the authors investigated the relationship between microvessel density of the resected nodules and the time-to-curve analysis. The study found that malignant nodules exhibited higher microvessel density, which correlated with the echo intensity change in the same nodules (r = 0.803, *p* < 0.001) [57] (Figure 3).

Another critical aspect in the IPMN classification is the involvement of the main pancreatic duct (MPD) [59]. This element is essential for defining the subtype of IPMN and guiding potential surgical action [60,61,62].

Evidence from surgical reports demonstrated that the enlargement of the MPD is not always due to an invasion of the duct but could be the result of ductal hypertension caused by mucin, protein plugs, or focal pancreatitis [59]. From previous experiences, preoperative radiological and pathological diagnoses of MPD involvement have a concordance of 70–75% [60,62]. A retrospective study evaluated the diagnostic accuracy of CH-EUS in MDP involvement in branch duct-type intraductal papillary mucinous neoplasms (BD-IPMNs), which are associated with a high risk of malignancy. A total of 166 patients with BD-IPMNs who underwent surgery were included, and CH-EUS was used to detect MPD involvement based on the presence of mural nodules. The CH-EUS findings were compared with pathological results. Sensitivity, specificity, and accuracy for MPD involvement detection using CH-EUS were 83.5%, 87.0%, and 84.9%, respectively. Among those with MPD involvement detected by CH-EUS, 71.6% had malignant lesions. Multivariate analysis identified MPD involvement on CH-EUS, among the significant risk factors for malignancy, together with age, cyst size, and mural nodule size [59].

## 5. CH-EUS in Pancreatic NET (pNET)

Neuroendocrine neoplasms (NENs) encompass a broad spectrum of pathologies that can exhibit manifold biological behavior, all of which share a common origin from diffuse neuroendocrine cells located virtually throughout the body [63].

However, the gastrointestinal (GI) tract is the most commonly affected site, and the global incidence of GI-NEN has been increasing sharply over the past 40 years [63,64], primarily as a result of an incidental diagnosis [65]. Of all GI NENs, pancreatic ones (pNENs) account for about 12% [66]. Traditionally, NENs are classified according to staging (dimensional), grading and the possible presence of a concomitant clinical syndrome related to the production and release of hormones by the mass (functioning and nonfunctioning tumors) [67]. Currently, it seems that grading, best defined by pathological analysis of Ki-67% and tumor differentiation, plays the central role in defining aggressiveness and predicting survival of NENs [68].

It should be noted, however, that NENs have a behavior that is sometimes difficult to understand, and although larger lesions may show local or vascular invasion, it is reported that even those <2 cm may show angioinvasion or lymph node metastasis; conversely, it is rarely reported that even G1 or G2 tumors may show distant metastasis making correct prediction very difficult [69].

For all NENs, it would be desirable to identify criteria independent of surgical specimen analysis that predict with good accuracy the risk of malignant behavior, and for pNENs it is even more important, in order to avoid, where possible, pancreatic surgery, which is burdened with high mortality and morbidity rates in favor of watchful waiting strategy [70,71].

EUS plays a key role in the diagnosis of pNENs [72] having also superior ability compared with CT to detect very small lesions [5].

EUS features recognized as associated with malignant behavior are hemorrhage and necrosis (appearing as hypoechoic and anechoic areas), hyalinosis, cystic changes, and a heterogeneous texture [73]. In contrast, the prognostic value of obstruction of the main pancreatic duct is more debated in the literature, with conflicting results [73,74,75].

It is well known from histopathologic studies of surgical specimens that reduced microvascular density (MVD) and increased intratumoral fibrosis are associated with more aggressive behavior of pNENs [76]. In the context of traditional radiology (CT, MRI), many studies show how radiological features correlate with histopathology, and in particular how certain vascular patterns at imaging correlate with reduced MVD and so becoming prognostic factors, associated with higher grading and presence of metastases [77,78]. Subsequently, it has been hypothesized that the same features could also be assessed by EUS, particularly when CE-EUS and CH-EUS are applied, allowing a magnification of tissue microvasculature [79] pNENs, including smaller ones, are usually hypervascular lesions at Doppler evaluation, and on both CE-EUS and CH-EUS typically appear hyperenhancing [21,80] making them easily diagnosed as compared to other typically malignant hypoenhancing solid pancreatic lesions such as PDAC and pseudopapillary solid tumor of the pancreas [20,81].

In 2010 the first study explored the utility of CE-EUS in predicting the behavior of pNENs. In a group of 41 patients with pathologically confirmed diagnosis of pNEN, three vascular patterns were arbitrarily defined: (1) diffuse enhancement, (2) filling defects and (3) no enhancement; the latter two patterns showed sensitivity, specificity and accuracy in detecting malignancy of 90.5%, 90.0% and 90.2%, respectively. In addition, the authors reported how the use of contrast improved the detection of other features indicative of advanced tumor, such as presence of areas of necrosis and hemorrhage (resulting in a contrast filling-defect pattern) with better sensibility compared to conventional B-mode [73].

A small case series on five patients with histologically established pNENs showed vasculature patterns at CE-EUS of persistent hypoenhancement, similar to that of adenocarcinoma, in a mixed lesion (NEN-adenocarcinoma), and a mixed vascular pattern (hyper- and hypoenhancement) in a G2 lesion, while in the other three G1 lesions hyper-enhancement was evident in early phase [82]

A more recent study in 2022 confirmed as previously reported that MVD predicts tumor aggressiveness: in the study, a lower MDV at histological report correlated with higher frequency of lymph node metastasis and G2-G3 grading; furthermore, in a subgroup of 36 patients who had previously been studied with CE-EUS, a low MVD showed a significantly higher frequency of arterial hypoenhancement and late washout (*p* = 0.042, *p* = 0.034) [83].

Role of CH-EUS in predicting the behavior of pNENs was explored by Palazzo et al. in 2018: evidence of heterogeneous enhancement during early arterial phase was defined as associated with pNEN aggressiveness (defined as G3 grading or presence of metastasis in G1/G2) [84]. Heterogeneous enhancement was found in 35 of 81 cases, corresponding to CH-EUS sensitivity, specificity, PPV, NPV and accuracy in predicting histopathological aggressiveness of 96%, 82%, 71%, 98%, and 86% respectively. In the comparisons of this study, it is relevant to underline that, even if the study was retrospective, the endoscopists were blinded to the patient’s history (including previous radiological examinations) and that CH-EUS was more accurate than Ki-67% and tumor size (>2 cm) for predicting aggressiveness in G1/G2 tumors. In addition, heterogeneous enhancement was confirmed to correspond to fewer vascular structures and more fibrotic structures upon pathological analysis [84].

A subsequent study of 47 lesions identified how the CH-EUS pattern of hypoenhancement correlated with pathologically established aggressiveness with sensitivity, specificity, and accuracy of 94.7%, 100%, and 97.9%, respectively, with better performances than prediction by contrast-enhanced CT (*p* < 0.001). It should be noted, however, that in this case some diagnoses were obtained on FNA (nonsurgical) specimens, and both neuroendocrine carcinomas and mixed neuroendocrine/non-neuroendocrine neoplasms were among the aggressive lesions. In the same study it is interesting to note that CH-EUS showed good correlation with histology (evidence of fibrosis and smaller, less numerous vessels) and correct predictive ability for poor prognosis in G1/G2 lesions [10].

Interestingly, another study matched OS and the 1-year survival rates of 39 patients with confirmed pNENs with three arbitrarily defined CH-EUS patters according on early and late-phase imaging: (1) vascular rich in both phases, (2) vascular rich and vascular poor in early and late phases, respectively, (3) vascular poor in both. Vascular poor pattern in both phases showed the shortest overall survival and the lowest 1-year survival rate, while the vascular-poor pattern on late-phase had the highest accuracy (89.2%) in predicting pNENs aggressiveness [85].

Next, Takada et al. studied TIC (time–intensity curves) parameters as potential predictors of pNEN aggressiveness (as compared to grading). Echo intensity change, decrease rate for enhancement, and enhancement ratio for node/pancreatic parenchyma showed high diagnostic performance, and after obtaining the cutoff value from the receiver operating characteristic (ROC), their diagnostic rates were 96.7%, 100%, and 100%, respectively, in distinguishing G1/G2 lesions from G3/neuroendocrine carcinoma. It should be noted, however, that the sensitivity in predicting the aggressiveness of G1/G2 lesions was lower in this setting, and measurements of TICs were not feasible in all patients [86]. In contrast, a more recent study in 2022 sought to establish an association between quantitative analysis of tumor vasculature (with parameters derived from TIC analysis obtained with VueBox software) and survival outcome after treatment in patients with pNENs or adenocarcinoma. In a multivariate model, the parameters analyzed did not highlight correlation with OS for pNENs, finding it instead for adenocarcinoma [87].

Finally, Yang et al. compared tumor grading with TIC parameters (obtained with the same VueBox software) and found that most values of low-grade lesions were higher than or equal to those of surrounding parenchyma, while most G3/pNECs showed lower TICs than both surrounding parenchyma and low-grade pNENs (*p* < 0.05). Notably, a cutoff value of less than 0.855 of the relative area under the curve was able to predict the distinction of G3/pNEC from G1/G2 with sensitivity, specificity and accuracy of 90.9%, 83.9% and 94.7% [88].

All studies reported so far show a correlation between endoscopic vascular patterns and prognosis, converging on the cardinal principle already known from histopathologic studies that a rarefied vasculature is generally synonymous with aggressive behavior [76]. However, the different studies describe similar but not analogous patterns, and there is no standardization. It should also be emphasized that these are studies with monocentric or at most bicentric retrospective design, with modest sample sizes, that not in all cases the comparison was made with surgical specimens (but on biopsy specimens), that the very definition of aggressiveness is not homogeneous, and that subsequent follow-up is often limited.

While CH-EUS has demonstrated a strong correlation with histopathologic features and prognosis, artificial intelligence (AI) applications in this field remain in their early stages [89]. To date, AI-based models for pNENs have primarily relied on conventional EUS images without contrast enhancement. A recent study by Mo et al. [90] successfully developed an ultrasomics-based model that achieved an AUC of 0.987 (95% CI: 0.965–1.000) in the training cohort and 0.781 (95% CI: 0.5933–0.9695) in the test cohort for predicting pNEN grading, demonstrating promising accuracy in classifying pNENs by grade. The model’s clinical utility was further supported by high decision curve analysis (DCA) and calibration curve scores, highlighting the potential of AI-driven image analysis. However, the integration of AI with contrast-enhanced imaging could further improve predictive ability. In this regard, Huang et al. [91] developed a deep learning model incorporating CE-US features, achieving an AUC of 0.81 (95% CI: 0.62–1.00) for predicting the aggressiveness of pNENs. When combined with clinical factors, such as tumor size and arterial enhancement, a nomogram was created that demonstrated an AUC of 0.85 (95% CI: 0.69–1.00) in the test cohort, with excellent calibration and clinical utility verified by decision curve analysis.

Moreover, further challenges may be provided by rarer histologies. For examples, pancreatic mixed adenoneuroendocrine carcinoma (pMANEC) is a rare and aggressive tumor composed of both ductal adenocarcinoma and neuroendocrine carcinoma components, each comprising at least 30% of the lesion. Its clinical behavior is typically driven by the more aggressive element, often the high-grade neuroendocrine component, and prognosis is generally poor. From a CH-EUS perspective, pMANEC presents a diagnostic challenge due to its dual vascular nature. The adenocarcinoma component usually appears as a hypoenhancing, poorly vascularized area, while the neuroendocrine component often shows hyperenhancement, due to its rich capillary network. As a result, CH-EUS may reveal a heterogeneous enhancement pattern within the same lesion, which is atypical for pure pancreatic adenocarcinoma or neuroendocrine tumors. This heterogeneity can raise suspicion for a mixed tumor and guide targeted biopsy. However, CH-EUS cannot confirm the diagnosis, which requires histopathological and immunohistochemical analysis for definitive characterization.

Given the superior resolution of CH-EUS compared to transabdominal CEUS, the application of AI-driven models to CH-EUS appears promising. In the future, one application of AI may be the automated analysis of time–intensity curves. As a matter of fact, AI algorithms can process large volumes of image data in real-time, accurately identifying regions of interest and extracting quantitative perfusion parameters such as peak intensity, time to peak, and wash-in/wash-out slopes. This may further reduce subjectivity and inter-observer variability, leading to more consistent assessments of lesion vascularity, particularly useful in differentiating malignant from benign pancreatic or lymph node lesions. Moreover, looking further, robotics-integrated EUS platforms can be enhanced with AI to support standardized probe positioning and image acquisition [90]. This integration improves reproducibility and ensures optimal imaging angles, which is crucial for high-quality TIC generation and interpretation. AI-powered robotics could also assist in lesion tracking and real-time guidance during procedures. By combining real-time TIC interpretation and robotic precision, AI has the potential to transform CH-EUS from a highly operator-dependent modality into a more standardized, accurate, and accessible diagnostic tool across diverse clinical settings.

## 6. Limitations

Contrast-enhanced harmonic endoscopic ultrasound (CH-EUS) offers valuable insights into the vascularity of pancreatic lesions, but several limitations still hinder its routine application. One of the main challenges is the high degree of operator dependency, as both image acquisition and interpretation require advanced technical skills and experience. This can lead to variability in diagnostic performance across different centers and users. Compounding this issue is the limited access to structured training programs and supervised hands-on experience, which may hinder the development of optimal proficiency in CH-EUS imaging interpretation, especially in low-volume or resource-limited settings. Additionally, assessing deeply located pancreatic lesions, particularly in obese patients, may result in suboptimal image quality due to limited spatial resolution, making it difficult to clearly delineate microvascular patterns. Another important limitation lies in the overlap of contrast enhancement features between certain benign and malignant lesions—such as chronic pancreatitis and pancreatic ductal adenocarcinoma—which can reduce specificity and complicate clinical decision-making. While CH-EUS has shown promising results in evaluating pancreatic adenocarcinoma, neuroendocrine tumors, and IPMNs, evidence supporting its utility in other types of pancreatic lesions, including metastases and lymphoma, remains limited. The use of contrast agents may not be suitable for all patients due to contraindications such as cardiac shunts or hypersensitivity, and their regulatory status and availability differ across regions. Moreover, the lack of standardized imaging protocols and interpretation criteria affects reproducibility and limits broader clinical adoption. Finally, although CH-EUS enhances lesion characterization, it does not provide tissue samples, necessitating fine-needle aspiration or biopsy for definitive diagnosis.

## 7. Conclusions

In conclusion, CH-EUS emerges as a highly valuable tool in the management of pancreatic lesions, including PDAC, IPMNs, and pNET. CH-EUS significantly improves the detection and characterization of pancreatic lesions, offering superior sensitivity and specificity compared to traditional imaging modalities. Its ability to assess tumor vascularity and microcirculation provides crucial insights into tumor aggressiveness, aiding in staging, predicting chemotherapy response, and evaluating the potential for surgical intervention. Although the role of CH-EUS may potentially expand to various types of lesions, such as lymphomas and metastases, available data on these applications remain limited. In the context of PDAC, CH-EUS enhances the accuracy of preoperative staging, detection of metastasis, and differentiation of malignant from benign lymph nodes, thus influencing clinical decision-making. Additionally, for IPMNs and pNET, CH-EUS plays a critical role in identifying high-risk features associated with malignancy, such as mural nodules, and in guiding management decisions. Overall, CH-EUS represents a promising advancement in the diagnostic and prognostic evaluation of pancreatic lesions, offering a more personalized and effective approach to patient care.

## List of Abbreviations

EUS: endoscopic ultrasound; IPMN: intraductual papillary mucinous neoplasm; PDAC: pancreatic ductal adenocarcinoma; CE-EUS: contrast enhanced endoscopic ultrasound; CH-EUS: contrast-enhanced harmonic endoscopic ultrasound; MVD: microvascular density; AI: artificial intelligence; NET: neuroendocrine tumors; NEN: neuroendocrine neoplasms; DFI-EUS: detective flow imaging; CT: computed tomography; MRI: magnetic resonance imaging; TIC: time–intensity curves; pMANEC: pancreatic mixed adenoneuroendocrine carcinoma.
